# *EAP1* regulation of GnRH promoter activity is important for human pubertal timing

**DOI:** 10.1093/hmg/ddy451

**Published:** 2019-01-04

**Authors:** Alessandra Mancini, Sasha R Howard, Claudia P Cabrera, Michael R Barnes, Alessia David, Karoliina Wehkalampi, Sabine Heger, Alejandro Lomniczi, Leonardo Guasti, Sergio R Ojeda, Leo Dunkel

**Affiliations:** 1 Centre for Endocrinology, William Harvey Research Institute, Barts and the London School of Medicine and Dentistry, Queen Mary University of London, London, UK; 2 Centre for Translational Bioinformatics, William Harvey Research Institute, Barts and the London School of Medicine and Dentistry, Queen Mary University of London, London, UK; 3 Centre for Integrative Systems Biology and Bioinformatics, Department of Life Sciences, Imperial College London, London, UK; 4 Children’s Hospital, Helsinki University Hospital and University of Helsinki, Helsinki, Finland; 5 Department of Pediatric Endocrinology, Children’s Hospital Auf der Bult, Hannover, Germany; 6 Oregon National Primate Research Center/Oregon Health and Science University, Portland, OR, USA

## Abstract

The initiation of puberty is orchestrated by an augmentation of gonadotropin-releasing hormone (GnRH) secretion from a few thousand hypothalamic neurons. Recent findings have indicated that the neuroendocrine control of puberty may be regulated by a hierarchically organized network of transcriptional factors acting upstream of GnRH. These include *enhanced at puberty 1* (*EAP1*), which contributes to the initiation of female puberty through transactivation of the GnRH promoter. However, no *EAP1* mutations have been found in humans with disorders of pubertal timing. We performed whole-exome sequencing in 67 probands and 93 relatives from a large cohort of familial self-limited delayed puberty (DP). Variants were analyzed for rare, potentially pathogenic variants enriched in case versus controls and relevant to the biological control of puberty. We identified one in-frame deletion (Ala221del) and one rare missense variant (Asn770His) in *EAP1* in two unrelated families; these variants were highly conserved and potentially pathogenic. Expression studies revealed *Eap1* mRNA abundance in peri-pubertal mouse hypothalamus. EAP1 binding to the GnRH1 promoter increased in monkey hypothalamus at the onset of puberty as determined by chromatin immunoprecipitation. Using a luciferase reporter assay, EAP1 mutants showed a reduced ability to *trans*-activate the GnRH promoter compared to wild-type EAP1, due to reduced protein levels caused by the Ala221del mutation and subcellular mislocation caused by the Asn770His mutation, as revealed by western blot and immunofluorescence, respectively. In conclusion, we have identified the first *EAP1* mutations leading to reduced GnRH transcriptional activity resulting in a phenotype of self-limited DP.

## Introduction

Puberty represents the remarkable transition from childhood to adult life with the attainment of reproduction and adult stature. The onset of puberty is triggered by the reactivation of the hypothalamic–pituitary–gonadal (HPG) axis through augmentation of the pulsatile release of gonadotropin releasing hormone (GnRH) from the hypothalamus, which in turn causes an increased pulsatile gonadotropin release from the anterior pituitary. Pubertal onset shows a large variability among populations, due to ethnicity, environment, nutrition and stress, (1–3) although previous studies indicate that 60–80% of the variance in pubertal timing can be explained by heritable factors (4,5). However, the precise mechanisms underlying the regulation of puberty onset are still unknown.

In 2000, Rampazzo *et al.* (6) identified an intronless gene containing a zinc (Zn) finger domain, mapped to 14q24.3 and provisionally named *C14ORF4*. Following functional annotation of *C14ORF4* that indicated its pivotal role in pubertal timing, it was renamed *enhanced at puberty 1* (*EAP1)* (7) and later was assigned the name of *interferon regulatory factor 2 binding protein-like (IRF2BPL)*. *EAP1* mRNA has been shown to increase in the hypothalamus of rats and non-human primates at the time of puberty, and *EAP1* deficiency led to delayed puberty (DP) and disrupted estrous cyclicity in both rodents (7) and non-human primates (8). *Eap1* codes for a nuclear transcription factor, characterized by a dual transcriptional activity; it both *trans*-activates the GnRH promoter, which facilitates GnRH secretion, and inhibits the preproenkephalin promoter, which represses GnRH secretion. Therefore, *Eap1* transcriptional activity facilitates the initiation of female puberty, in a manner that is independent of hypothalamic Kiss1 expression (9). Notably, a study performed in rats showed that hypothalamic expression of *Eap1* is not directly regulated by ovarian steroids, as its expression in the peri-pubertal female hypothalamus changes even in the absence of ovaries (10). Despite this seemingly important role, no *EAP1* mutations have yet been identified in humans with pubertal disorders.

DP is defined as the absence of testicular enlargement in boys or breast development in girls at an age that is 2 to 2.5 standard deviations (SDs) later than the population mean (11). In absence of any identifiable cause, DP usually resolves by the age of 18 years, and in this case is referred to as self-limited, or constitutional, DP (12). Self-limited DP is commonly familial and segregates with an autosomal dominant inheritance patterns in >70% of families, indicating a strong genetic basis of the trait. Such an inheritance pattern suggests that DP has a monogenic or oligogenic background, although very few underlying genes have been discovered (13). Our large, well-phenotyped Finnish DP cohort (14) offers a prodigious tool to investigate potential pathogenic variants causing self-limited DP, likely enriched in our cohort. Indeed, comparable next generation sequencing strategies in patients from this cohort have unveiled roles for novel genes in the pathogenesis of self-limited DP (15,16).

In this study we have identified, for the first time, two *EAP1* mutations leading to reduced GnRH transcriptional activity resulting in the phenotype of self-limited DP.

## Results

### Exome sequencing of families with self-limited DP identifies variants in *EAP1*

**Figure 1:**
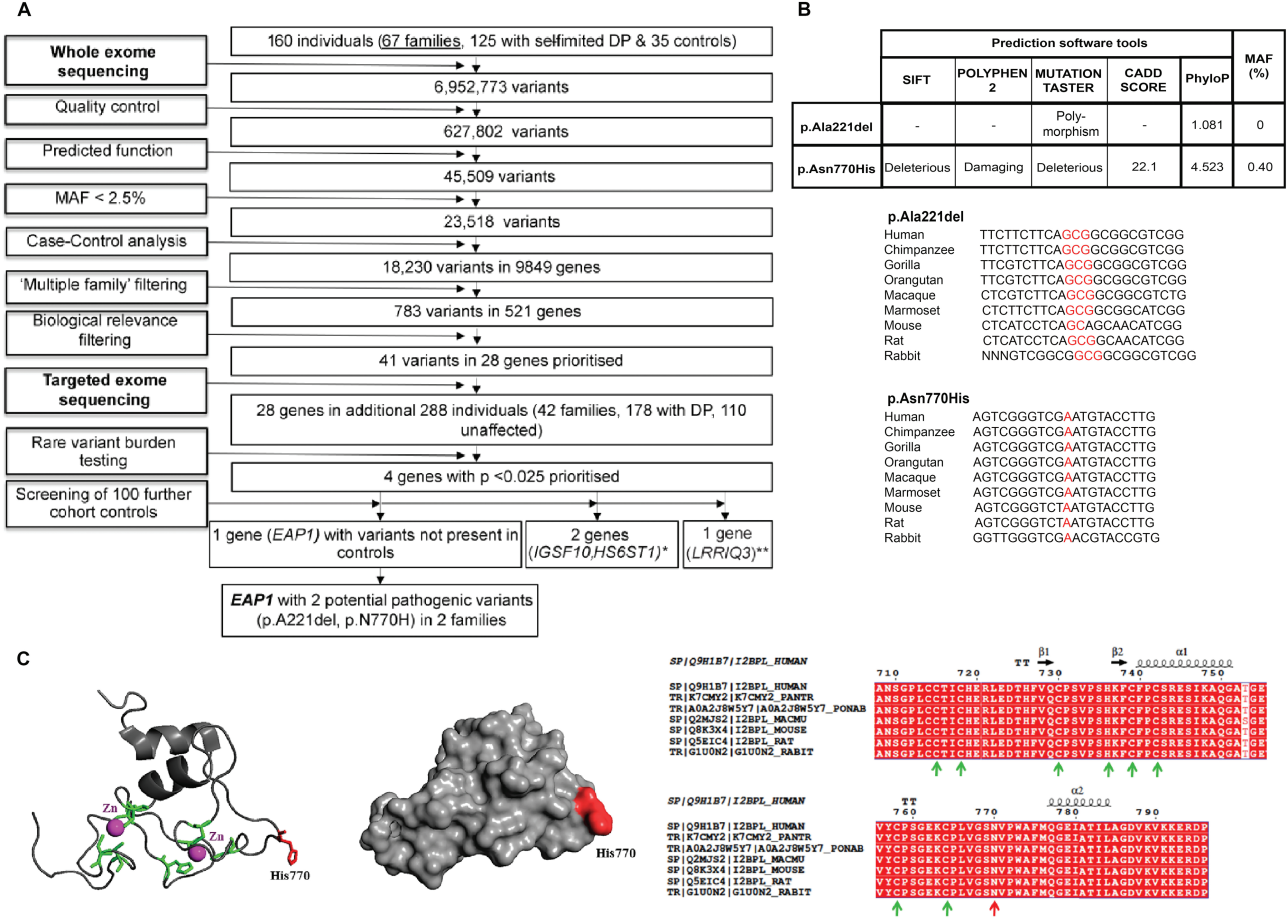
Filtering strategy to identify *EAP1* as a candidate gene for self-limited DP, prediction of pathogenicity and conservation across species for *EAP1* variants identified, and structural model of the Zn finger domain of EAP1. **(A)** WES was performed on DNA extracted from peripheral blood leukocytes of 160 individuals from our cohort (67 DP probands, 58 DP relatives and 35 controls). The exome sequences were aligned to the UCSC hg19 reference genome. Picard tools and the genome analysis toolkit were used to mark PCR duplicates, realign around indels, recalibrate quality scores and call variants. Variants were filtered for potential causal variants using filters for quality control, predicted functional annotation, MAF, case–control analysis, variants in multiple families and biological relevance. Targeted exome sequencing using a Fluidigm array of 28 candidate genes identified post-filtering was then performed in a further 42 families from the same cohort (288 individuals, 178 with DP and 110 controls). Variants post-targeted resequencing were filtered using the same criteria as the WES data. RVBT was performed with a multiple comparison adjustment applied post*-hoc* (17). Screening of 100 further cohort controls was via conventional Sanger sequencing. ^*^Data published (15, 16). ^**^Excluded due to the presence of variants in multiple controls. **(B)** Minor allele frequencies for ExAC Finnish population (accessed February 2018), conservation and pathogenicity scores (SIFT (37); Polyphen2 (36); MutationTaster (46)). Multiple sequence alignment (msa) was generated using MutationTaster (46). The p.Ala221 residue is highly conserved among different species, PhyloP score 1.801. The p.Asn770His is highly conserved among different species and the PhyloP score is 4.523. **(C)** The mutant 3D structure of the Zn finger domain of EAP1 is presented as a cartoon. The Zn atoms are presented as magenta spheres, the conserved C3HC4 residues (which bind Zn atoms) are presented in green and the mutant histidine (H) at position 770 is presented in red. The mutant His770, shown in red, is located on the surface of EAP1 and may be part of a protein–protein interaction site. The position of invariable residues C3HC4 is indicated with green arrows, whereas the position of N770 is indicated with a red arrow. The amino acid numbering and secondary structure is presented above the msa. C indicates cysteine.

Whole-exome sequencing (WES) of 67 informative families from our large cohort with self-limited DP returned 6 952 773 variants (Fig. 1A). These variants were filtered through our in-house pipeline to identify rare, predicted deleterious mutations, segregating in multiple families and with potential biological relevance. The 28 top candidate genes identified then underwent targeted resequencing in additional 42 families from the same cohort (178 individuals with DP and 110 controls), and the filtered results were analyzed by applying statistical thresholds for enrichment of rare, pathogenic variants in our cohort via whole-gene rare variant burden testing (RVBT) with multiple comparison adjustment (17). Four genes passed the *P* < 0.025 threshold on RVBT, and potentially pathogenic variants in these genes were further analyzed to determine their presence in controls from our cohort and for segregation with trait (Fig. 1A).

The candidate gene, *EAP1* (ENSG00000119669; gene identification number 64207; synonyms: *IRF2BPL* and *Chromosome 14 Open Reading Frame 4 [c]*), was identified after RVBT (adjusted *P* = 1.99E-05). *EAP1* has been highlighted as an important potential candidate for the hierarchical regulation of pubertal timing through systems biology approaches and animal models (18). *EAP1* mRNA levels and protein expression are seen to increase in the hypothalamus of primates and rodents at the time of pubertal onset (7).

### Two identified variants in *EAP1* are rare, highly conserved and potentially damaging to protein function

Eight rare and potentially pathogenic variants were identified in *EAP1* from whole and targeted exome sequencing. Five of these variants were discarded in our post-sequencing analysis, as they were present in multiple controls from our cohort. Three variants of interest in *EAP1* were initially identified in four pedigrees from the cohort, but only two were found to segregate within families after Sanger sequencing of all family members. These two variants [NM_024496.3: c.2308A>C (rs760847179) p.Asn770His Chr: 14:77491828 and NM_024496.3: c.661_663delGCG p.Ala221del Chr: 14:77493473] were each identified in one proband from the cohort and their affected family members. The two probands from these pedigrees did not carry any other predicted pathogenic variants in known GnRH deficiency or gonadotropin deficiency-causing genes (16). The two *EAP1* variants, p.Asn770His and p.Ala221del, are both rare with a minor allele frequency (MAF) of <0.5% in population databases. Both variants affect amino acids that are highly conserved among homologues, as revealed by PhyloP score, and multiple sequence alignment (Fig. 1B). The p.Asn770His missense variant is predicted to be deleterious to protein function by prediction tools and CADD score, as the affected amino acid residue resides in a long loop region, which is C-terminal to the C3HC4 ring domain (residues 715-762). This region shows evolutionary conservation among different species (Fig. 1C). Although the amino acid substitution (p.Asn770His) does not affect the residues directly involved in Zn binding and it is not predicted to reduce protein stability, it introduces a positively charged residue in place of a neutral one at a potential protein–protein interaction site and is thus likely to be disruptive. The p.Ala221del is a novel deletion located within a predicted disordered and glycine–proline-rich region of the protein, but unfortunately no structural information could be obtained.

### Family members with classical self-limited DP from two pedigrees carry heterozygous *EAP1* variants inherited in an autosomal dominant pattern

**Figure 2:**
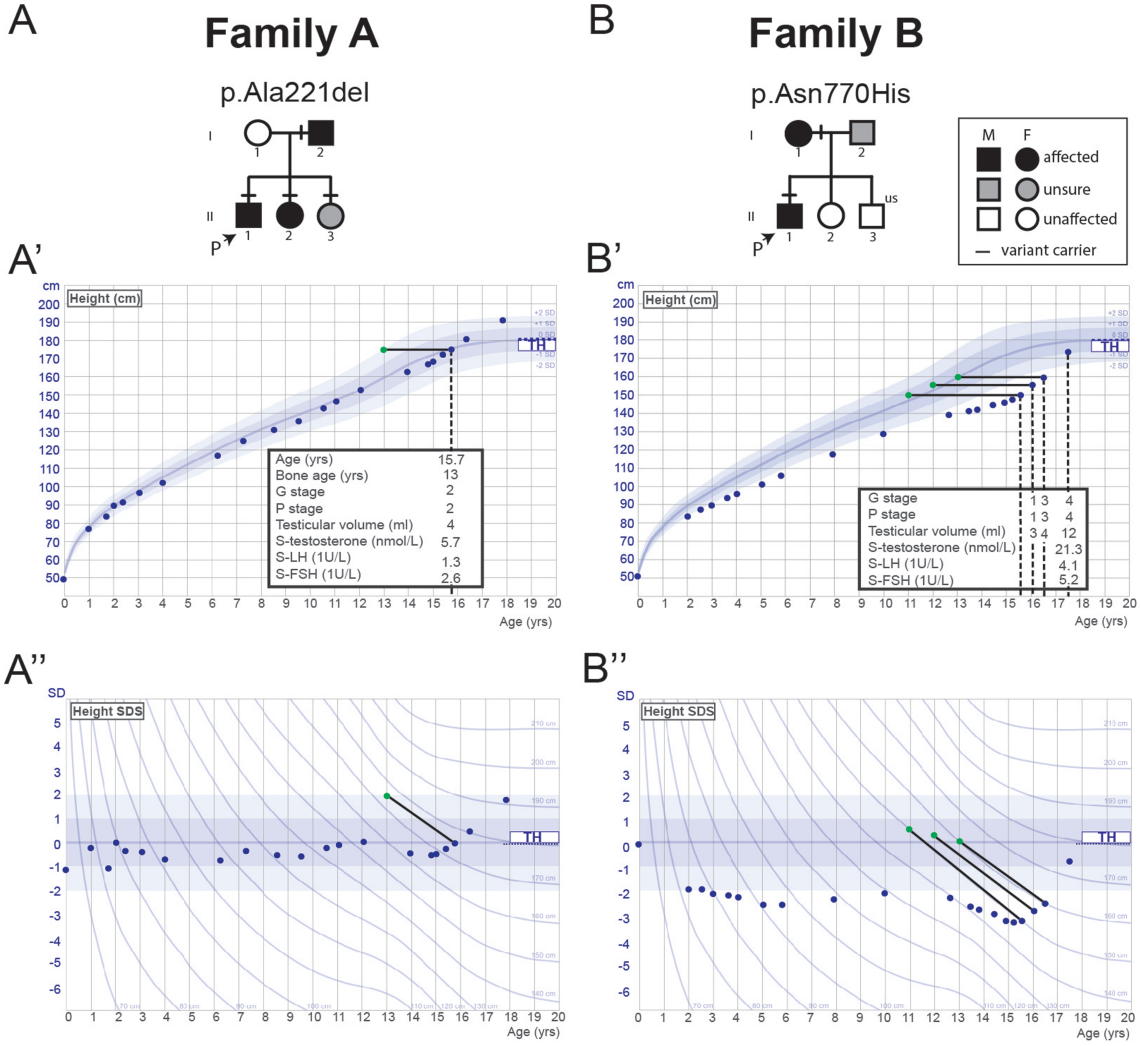
Pedigrees of the families with *EAP1* mutations with proband growth charts. **(A and B)** Squares indicate male family members and circles indicate female family members. Black symbols represent clinically affected, gray symbols represent unknown phenotype and clear symbols represent unaffected individuals. ‘P’ indicates the proband in each family and ‘us’ indicates un-sequenced due to lack of DNA from that individual. A horizontal black line above an individual’s symbol indicates they are heterozygous for that mutation as confirmed by either WES or Fluidigm array and verified by Sanger sequencing. **(A′**, **A^″^**, **B^′^** and **B″)** Height and height SDS charts for the probands of each of the two pedigrees. Tanner genital stage (G stage), Tanner pubic hair stage (P stage), testicular volume, standardized (S)-testosterone, LH and FSH values are given for each proband at various time points. Normal values, based on data from >70 000 healthy Finnish children, have been previously published (15). TH means target height based on mid-parental heights. Green dots connected by continuous black lines indicate bone age at the corresponding chronological age (blue dots), as estimated by the Greulich and Pyle method.

Segregation with a clear autosomal dominant pattern of inheritance was seen in both the identified families, with the affected individuals carrying heterozygous changes in *EAP1* variants (Fig. 2A and B). The affected individuals from these two families have classical clinical and biochemical features of self-limited DP, with delayed onset of Tanner stage II and delayed peak height velocity (PHV) (Fig. 2A′, A^″^, B′ and B^″^). The proband from family A presented at the age of 15.7 years with delayed pubertal development. His father had had similar delay in puberty onset. At initial evaluation the proband had testes volumes of 4 ml and circulating testosterone concentration at early pubertal level (5.7 nmol/l). The proband from family B presented at the age of 15.3 years with pre-pubertal testes volumes of 3 ml bilaterally. Both probands had markedly delayed bone age at presentation and during follow-up they had spontaneous pubertal development without testosterone therapy excluding idiopathic hypogonadotropic hypogonadism.

### *Eap1* is expressed within key regions within the mouse adult hypothalamus

**Figure 3:**
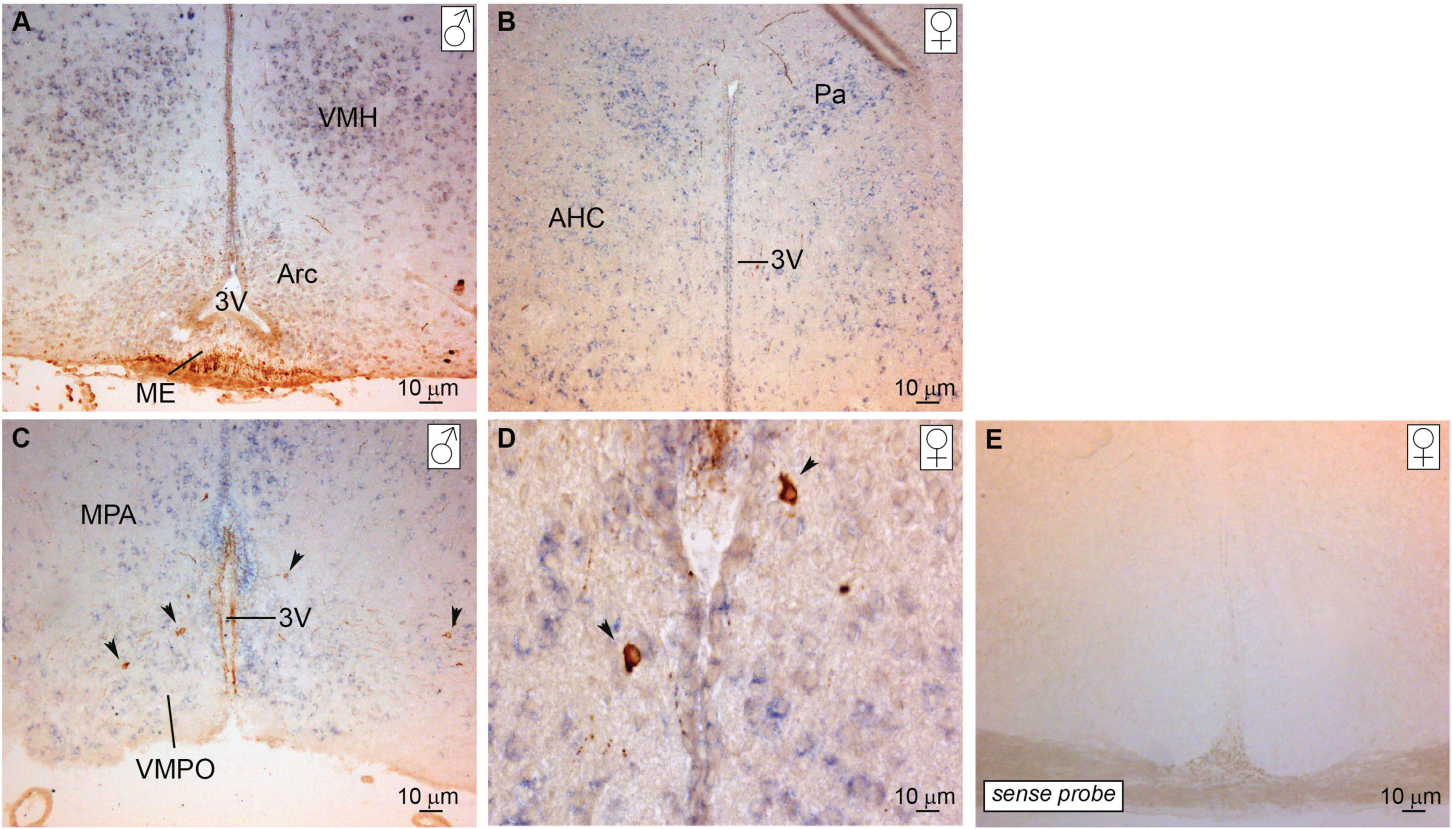
*Eap1* mRNA is expressed in the hypothalamus of peri-pubertal mice. Peri-pubertal mice hypothalamus sections were used for *in situ* hybridization and immunohistochemistry to localize *Eap1* mRNA (staining in purple) and GnRH neurons (staining in brown), respectively. *Eap1* is expressed in the VMH, Arc and Pa nuclei of **(A)** male and **(B)** female mice. GnRH neuron bodies are dispersed predominantly in the medial preoptic area **(C)** in an *Eap1* positive milieu. GnRH neuron bodies are shown at higher magnification in **(D)**. GnRH neuron projections are also detected in the ME (A) and at the level of the 3V (C). Sense probe was used as a negative control with no detectable staining **(E).** Arrows indicate GnRH neuron bodies. ♀ indicates female; ♂, male; 3V, third ventricle; AHC, anterior hypothalamic area, central part; MPA, medial preoptic area; VMPO, ventromedial preoptic nucleus.

We performed *in situ* hybridization on peri-pubertal hypothalamus of male and female mice. An abundance of *Eap1* mRNA was detected in the ventromedial (VMH), paraventricular (Pa) and arcuate (Arc) nuclei of male (Fig. 3A) and female (Fig. 3B) mice. *Eap1* expression was also investigated concurrently with the detection of GnRH neurons using immunohistochemistry. GnRH neuronal axons were mainly detected at the level of the median eminence (ME; Fig. 3A), whereas GnRH neuron bodies were interspersed in the median preoptic area in a positive *Eap1* milieu (Fig. 3C; Fig. **3**D image shows GnRH neuron bodies at high magnification). *Eap1* expression specificity was tested with a sense probe (Fig. 3E), which resulted in no detectable staining. Hence, *Eap1* displayed strong expression in specific hypothalamic subregions of both female and male peri-pubertal mice. These results in mice are in keeping with *Eap1* expression in rat and non-human primate hypothalami (7). Previous evidence showed GnRH neurons expressing Eap1 (7).

### EAP1 binding to the GnRH promoter increases at puberty

**Figure 4:**
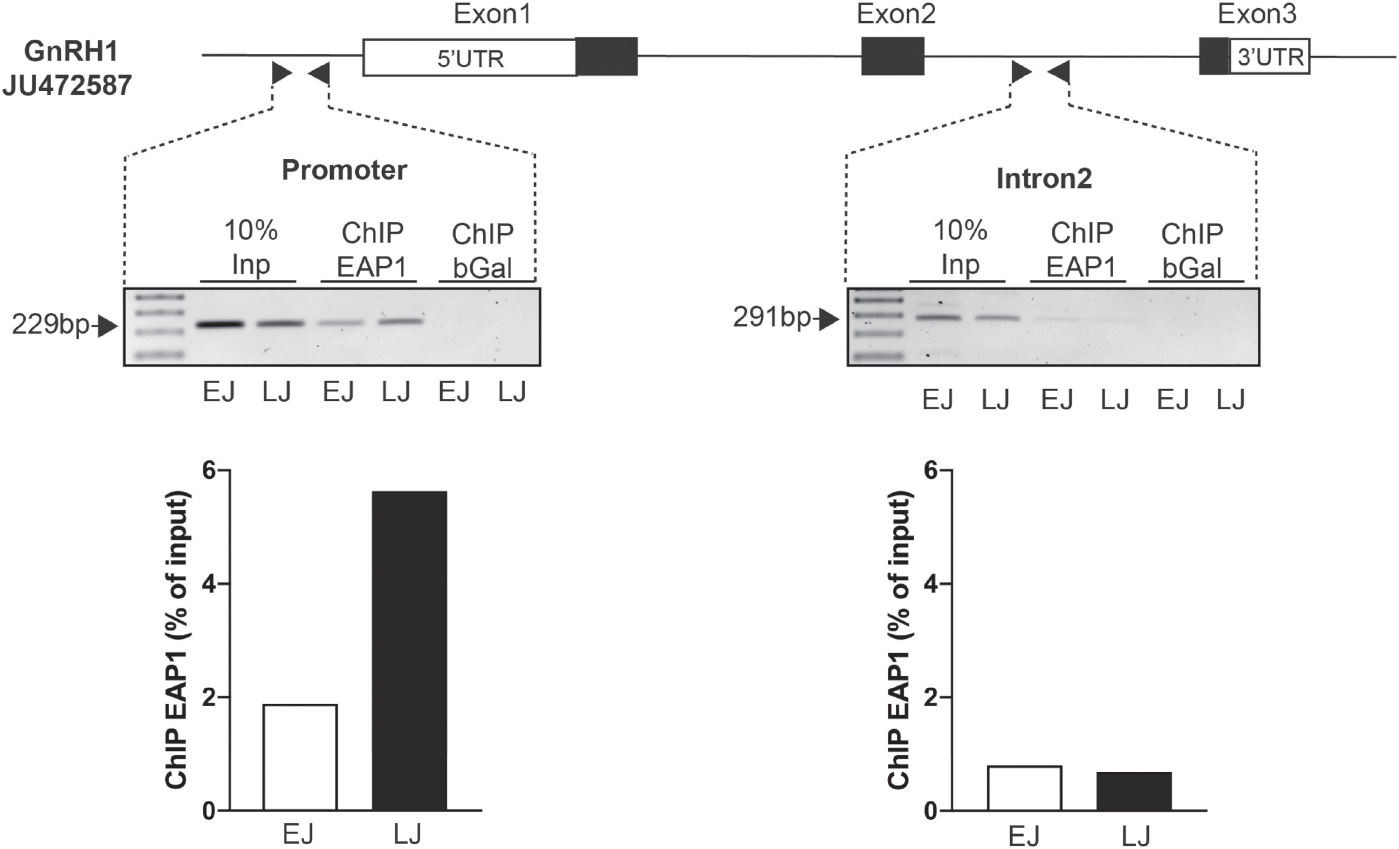
EAP1 binding to the GnRH promoter increases at puberty of female rhesus monkeys. An increased association of EAP1 to the rhesus GnRH1 promoter region in the MBH of female monkeys between the EJ and LJ periods is demonstrated. No change in EAP1 binding is detected in intron 2 of the GnRH gene. Inp means Input DNA and bGal means ChIP performed using an antibody to beta-galactosidase (a protein not present in the brain), serves as a negative control. Experiment was performed once, due to the limitation of availability of primate tissue.

Results of a chromatin immunoprecipitation (ChIP) experiment indicated that EAP1 binds to GnRH1 promoter (Fig. 4) and that association of EAP1 to the GnRH1 promoter region in the medial basal hypothalamus (MBH) of female monkeys is increased at the late juvenile (LJ), as compared to the early juvenile (EJ) period, i.e. at the initiation of puberty. No change in EAP1 binding was detected in intron 2 of the GnRH gene. ChIP also performed using an antibody to beta-galactosidase (not expressed in brain tissue) did not show any appreciable signal in either EJ or LJ animals.

### EAP1 variants significantly impair GnRH promoter activity in a dose-dependent manner

**Figure 5:**
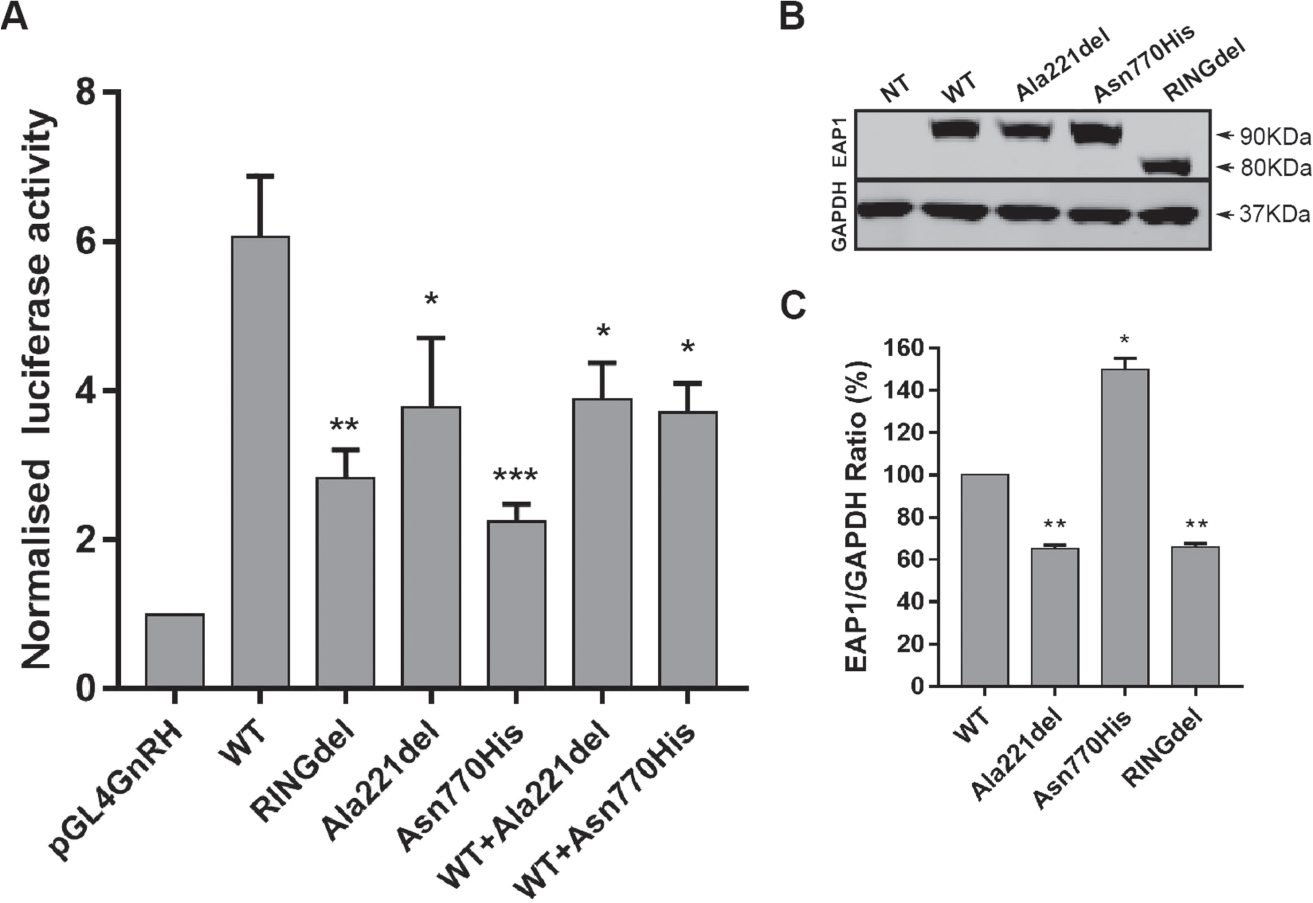
EAP1 mutations impair the transcriptional activity of the human GnRH promoter. **(A)** HEK293T cells were seeded at 17.5 × 10^4^ cells/well onto a 24-well plate and transiently transfected with *EAP1* plasmids (WT and mutated; 200 ng/well). Forty-eight hours post-transfection DLR assay was performed. Each transfection was normalized by co-transfecting with Renilla SV-40 vector and was performed in triplicate. *EAP1 trans*-activating GnRH promoter activity is significantly reduced by the in frame deletion (pAla221del) and missense (Asn770His) mutants compared to the WT. The mutants also cause a dose-dependent reduction of EAP1 WT transcriptional activity. The ablation of the RING finger domain (RINGdel) impairs the ability of *trans*-activating GnRH promoter and was used as a control, *n* = 3. **(B)** HEK293T cells were seeded at 0.3 × 10^6^ cells/well onto a 6-well plate and transiently transfected with *EAP1* plasmids (WT and mutated; 1 μg/well). Forty-eight hours post-transfection western blot analysis was performed. Eap1 protein expression is detected at the expected molecular weight (90 kDa) and RINGdel expression is detected at ~80 kDa, as a result of the deletion of the RING finger domain (amino acids 715-762). GAPDH was used as loading control and detected at 37 kDa. HEK293T cells do not express EAP1, *n* = 3. **(C)** Quantification of western blot analysis, indicating that Ala221del protein levels are significantly reduced and Asn770His protein levels are significantly increased.

Previous results (7), together with evidence from our ChIP analysis, demonstrate that *EAP1* transcriptional activity is promoter specific. We therefore employed a promoter assay in order to study GnRH promoter activity when *trans*-activated by EAP1 WT or mutated constructs (Fig. 5A). In a HEK293T cell line, the *trans*-activating strength of EAP1 on the human GnRH promoter was significantly reduced by the p.Ala221del in frame deletion variant (adjusted *P* = 0.0374) and highly significantly reduced by the p.Asn770His missense variant (adjusted *P* = 0.0003), as compared to the WT protein. The deletion of the RING finger domain (RINGdel), which is required for *EAP1* transcriptional activity (7), impairs the ability of EAP1 to *trans*-activate the GnRH promoter (adjusted *P* = 0.00021) and was used as a control (Fig. 5A)*.* In addition, as these are heterozygous mutations in our human patients, we co-transfected in equal amount the EAP1 WT expression vector with either the EAP1 Ala221del or with EAP1 Asn770His vectors, and we observed a dose-dependent reduction of EAP1 transcriptional activity (WT versus WT+Ala221del, adjusted *P*=0.0498; WT versus WT+Asn770His, adjusted *P*=0.0305).

### EAP1 mutant proteins levels are altered compared to wild-type protein

To further understand the mechanism by which these mutations affect protein function, we examined protein expression in a HEK293T cell line, which has no endogenous *EAP1* expression (Fig. 5B). EAP1 WT and mutated proteins were expressed (Fig. 5B) at the expected molecular weight of 90 kDa, while the RINGdel protein was expressed at 80 kDa, as expected due to the deletion of amino acids 715-762 (7). Densitometry analysis of the immunoblot (Fig. 5C), revealed that the Ala221del mutant protein levels, normalized to the housekeeping gene *glyceraldehyde-3-phosphate dehydrogenase* (*GAPDH*), are significantly reduced compared to WT (adjusted *P* = 0.0062), thus explaining the reduced activity of this mutant protein. However, the Asn770His mutant protein levels are increased significantly (adjusted *P* = 0.0322).

### The EAP1 Asn770His mutant protein is mislocated in the cytoplasm

**Figure 6:**
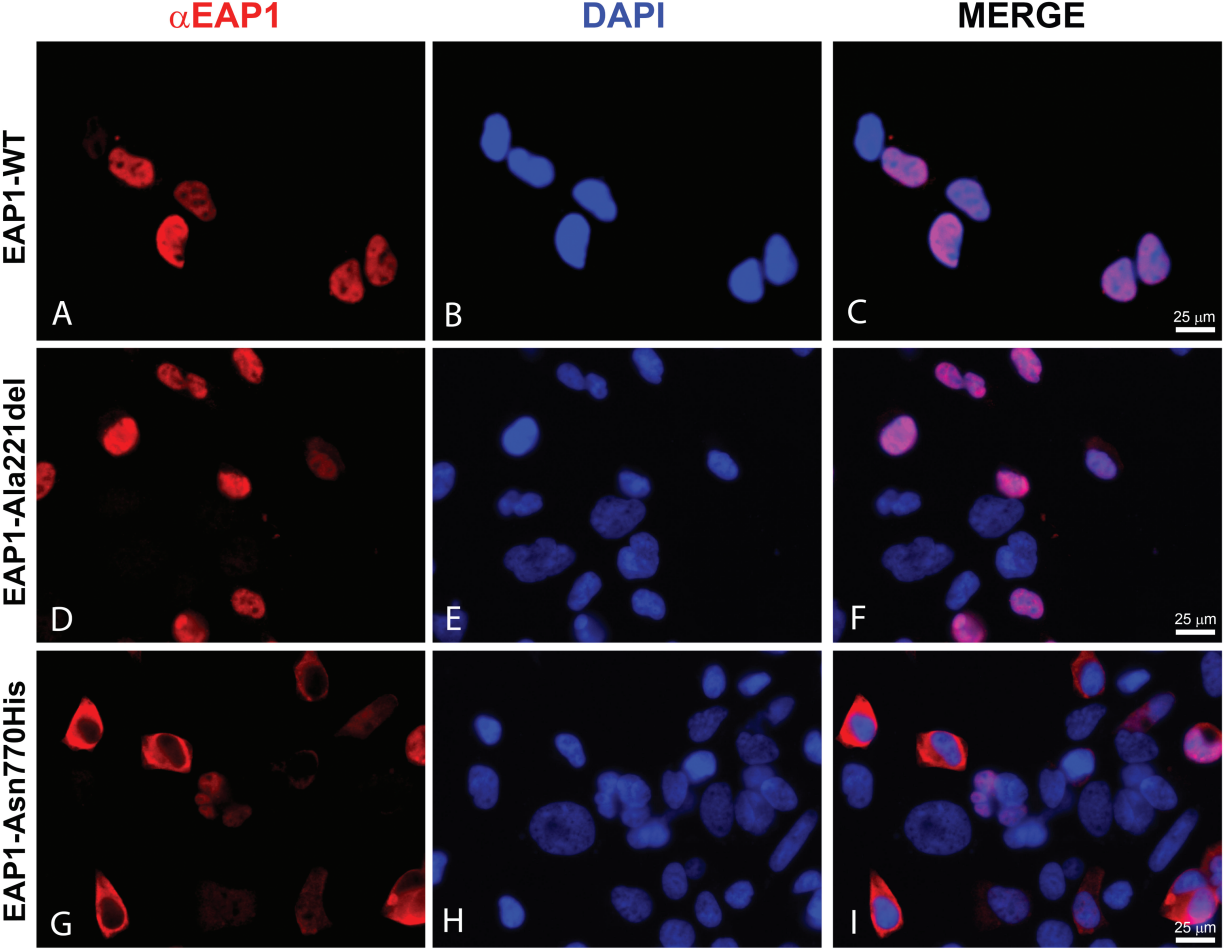
EAP1 p.Asn770His mutant is subcellularly mislocated. Immunofluorescence staining of EAP1 WT and mutated proteins (red) and DAPI (blue) in HEK293T cells. **(A**, **B and C)** show EAP1 WT protein expression within the nucleus, co-localizing with DAPI. **(D**, **E and F)** show EAP1 Ala221del mutant protein expressed within the nucleus, co-localizing with DAPI. **(G**, **H and I)** show EAP1 Asn770His mutant protein not expressed in the nucleus. Images were acquired using a fluorescence microscope (Leica microsystem, Germany) and processed using Adobe Photoshop CS6, *n* = 2.

EAP1 is a transcriptional factor that localizes to the nucleus. To determine whether the Ala221del or Asn770His mutations affect the ability of EAP1 to reach the cell nucleus, we performed an immunocytoflourescence analysis (Fig. 6) on HEK293T cells transiently transfected with EAP1 WT, Ala221del or Asn770His mutant vectors. The results showed that both the EAP1 WT protein and the Ala221del mutant are expressed in the nucleus, as evidenced by co-expression with the nuclear marker 4′,6-diamidino-2-phenylindole (DAPI; Fig. 6C and F). In contrast, the Asn770His mutant protein failed to reach the nucleus, remaining in the cytoplasm (Fig. 6I), hence providing a compelling explanation for the reduced functional activity of this mutant.

## Discussion

The central control of pubertal onset, after a period of juvenile quiescence, is mediated by a resurgence of the GnRH pulse generator, with a profound increase in the activity of the HPG axis. Although kisspeptin secretion from KNDy neurons in the Arc nucleus is one of the most important stimulatory elements of this neural network, kisspeptin has not been demonstrated as the ultimate controller of the release of the puberty brake but instead is likely to act as a conduit for upstream regulators (19). A growing body of evidence has demonstrated that no single gene alone is likely to be responsible for hypothalamic control of puberty. Instead, a sophisticated network of transcriptional factors, hierarchically organized, has been proposed as the machinery governing the balance between inhibitory and excitatory upstream inputs on the GnRH system (20). Transcriptional repression is a fundamental component of the regulation of mammalian gene expression, and transcriptional repressors containing Zn finger motifs, which recognize specific DNA sequences in regulatory regions of the genome, are particularly appealing candidates to have major roles in this governing network (21).

In this study we have identified through whole and targeted exome sequencing two deleterious mutations in *EAP1* in pedigrees with self-limited DP. *Eap1* had been proposed in several animal studies to have an important role in regulating the time of puberty onset; however, mutations in this gene have not, to our knowledge, been previously identified in conditions of abnormal pubertal timing in humans. *EAP1* mutations were significantly enriched in our cohort of patients with self-limited DP as compared to control populations, and these mutations were inherited in the expected autosomal dominant pattern seen in this condition (14,22). The affected members of these families displayed typical self-limited DP with late onset of puberty but full adult development by the age of 18 years. None of the affected individuals had neurological or other associated phenotypic abnormalities (23). Complementing previous studies in rats and non-human primates (7), we have shown that *Eap1* is also abundantly expressed in the peri-pubertal mouse hypothalamus. Previously published work provides evidence that GnRH neurons express *Eap1* (7). Moreover, we have demonstrated that EAP1 binding to the GnRH1 promoter increases at the onset of puberty in female monkeys. In a previous rodent model siRNA-mediated *Eap1* knockdown was shown to cause delayed vaginal opening in female rats in addition to disrupted estrous cyclicity; reduced plasma luteinising hormone (LH), follicular stimulating hormone (FSH) and estradiol levels; and delayed growth of ovarian follicles (7). In non-human primates knockdown of *EAP1* expression in the Arc lead to cessation of menstrual cyclicity, and a single-nucleotide polymorphism in the 5′-flanking region of the *EAP1* gene was associated with increased incidence of amenorrhea (8,24). EAP1 has been shown to *trans*-activate the GnRH promoter but reduce *Kiss1* transcription (25). This evidence together highlights the role of *EAP1* in regulating GnRH expression and suggests that *EAP1* may represent one of the main transcription factors contributing to the neuroendocrine control of female puberty.

Both of the mutations identified here in humans with DP led to an impaired ability of the mutant EAP1 protein to *trans*-activate the human GnRH promoter, with a dose-dependent reduction in protein function seen in the *in vitro* heterozygous model (of 50% wild-type and 50% mutant protein). Interestingly, one of the mutations identified led to reduced protein function secondary to decreased expression of the EAP1 protein, while the second resulted in mislocalization of the mutant protein to the cytoplasm.

Disturbances in pubertal timing affect over 4% of adolescents and are associated with adverse health outcomes. Specifically, DP patients are at risk of decreased bone mineral density, osteoporosis (26,27) and psychological distress (28,29). In addition, recently associated increased risk has been shown for outcomes such as cervical cancer, myocardial infarction and poor overall health (12,30). Thus, the genetic control of human puberty is not only a fascinating scientific puzzle but also has potential for major health impact for patients with abnormal pubertal timing, both precocious and delayed. Moreover, as the understanding of the genetic basis of both self-limited and other causes of DP improves, it is likely that genetic testing will be able to help to establish a definitive diagnosis in cases, particularly where there is diagnostic difficulty. Rapid and efficient diagnosis of patients in clinic would represent a significant advancement in patient care and a likely economic advantage.

In summary, we have identified two pathogenic mutations in the central transcriptional regulator *EAP1* as the likely cause for self-limited DP in two families. Mutations in several genes known to influence GnRH secretion have been identified in patients with pubertal delay, including *TAC3*, *TACR3*, *KISS1* and, now, *EAP1* (31–33). Our results strengthen the concept that *EAP1* is a bona fide regulator of pubertal onset and add to the understanding of the regulatory neural network that controls the onset of human puberty.

## Materials and Methods

### Patients

The cohort of individuals (*n* = 910) we studied here has been described in previous reports (14–16). Briefly, the cohort includes patients with self-limited DP (*n* = 492), defined as the onset of Tanner genital stage II (testicular volume, >3 ml) >13.5 years in boys or Tanner breast stage II >13.0 years in girls (i.e. 2 SD later than average pubertal development) (11) and their unaffected relatives. The patients were referred with a diagnosis of self-limited DP to a specialist pediatric care in Finland between 1982 and 2004. All patients met the diagnostic criteria for self-limited DP; medical history, clinical examination and routine laboratory tests were reviewed to exclude those with chronic illness. Hypogonadotropic hypogonadism, if suspected, was excluded by spontaneous pubertal development at follow-up.

Family members of the DP patients participated via structured interviews and using archived height measurement records. The criteria for DP in probands’ family members were (i) age at take-off or (ii) PHV occurring 1.5 SD beyond the mean (i.e. age at take-off exceeding 12.9 and 11.3 years or age at PHV exceeding 14.8 and 12.8 years in males and females, respectively) or (iii) age at attaining adult height more than 18 and 16 years, in males and females, respectively (34). Written informed consent was obtained from all participants. The study protocol was approved by the ethics committee for Pediatrics, Adolescent Medicine and Psychiatry, Hospital District of Helsinki and Uusimaa (570/E7/2003). UK ethical approval was granted by the London-Chelsea National Research Ethics Service (NRES) committee (13/LO/0257). The study was conducted in accordance with the guidelines of the Declaration of Helsinki.

### Genetic analysis

Genetic analysis was performed in 67 probands with DP, from those 67 families with the greatest number of affected individuals in our cohort (male, *n* = 57; female, *n* = 10), 58 affected family members (male, *n* = 36; female, *n* = 22) and 35 of their unaffected family members (male, *n* = 13; female, *n* = 22). WES was performed on DNA extracted from peripheral blood leukocytes of these 160 individuals, using a Nimblegen V2 or Agilent V5 platform and Illumina HiSeq 2000 sequencing. The exome sequences were aligned to the UCSC hg19 reference genome using the Burrows–Wheeler Aligner software [BWA-MEM (bwa-0.7.12)]. The software Picard tools (picard-tools-1.119) was used to sort alignments and mark PCR duplicates. We used the genome analysis toolkit (GATK-3.4-46) to realign around indels and recalibrate quality scores using dbSNP, Mills and 1000 Genomes as reference resources. Variant calling and joint genotyping using pedigree information were performed using HaplotypeCaller in GVCF mode from the genome analysis toolkit. The resulting variants were filtered using the variant quality score recalibration from GATK.

Variants were analyzed and filtered for potential causal variants using filters for quality control, predicted function, MAF and biological relevance (Fig. 1A). Filtering by MAF included only variants with MAF <2.5% in the 1000 Genomes database, the NHLBI exome variant server and the Exome Aggregation Consortium (ExAC) and gnoMAD databases. Biological relevance filtering allowed prioritization of variants in genes with potential biological significance in the control of pubertal timing, using tools including Ingenuity Variant Analysis (QIAGEN Redwood City), Genego MetaCore (Thomson Reuters), OMIM, UniProt and Annovar (35). The multiple family filter retained only genes with variants present in more than one proband, and the case–control analysis excluded variants present in more than one unaffected control from our Finnish cohort, represented by family members with timing of puberty within the normal range. Targeted exome sequencing (Fluidigm) of the remaining candidate gene post-filtering was performed in a further 42 families from the same cohort [288 individuals, 178 with DP (male, 106; female, 72) and 110 controls (male, 55; female, 55); Fig. 1A], with filtering as in (15). Whole-gene RVBT was performed post-sequencing. Fisher’s exact test was used to compare the prevalence of deleterious variants in our cohort with a set of controls from the Finnish population (ExAC European Finnish), taken from the ExAC Browser (ExAC, Cambridge, MA: accessed September 2015). For each gene, all variants from the ExAC database with a MAF of <2.5%, predicted to be deleterious by both Polyphen-2 (36) and SIFT (37), were included in the analysis, with each family in our cohort represented by the proband only. A multiple comparison adjustment was applied post-*hoc* using the Benjamini and Hochberg method (17), as detailed in (15).

### *In silico* analysis

The mutant 3D structure of EAP1 harboring the Asn770His variant was obtained using Phyre2 program (38) and the mutant EAP1 FASTA sequence. Protein disorder was predicted using the DISOPRED3 server (39). The human experimental structure of EAP1 (PDB: 2cs3) was used as template.

### Site-directed mutagenesis

*EAP1* mutations were inserted in pcDNA3.1/Zeo-h*EAP1* (7) using QuikChange II Site-Directed Mutagenesis Kit (Agilent Technologies) following the manufacturer’s instructions. The p.Ala221del in-frame deletion was inserted using the following primers: forward 5′-GCGGCGTCGGTGGCGTCTCGGCGTGGAAC-3′ and reverse 5′-GCCACCGACGCCGCTGAAGAAGAATTGGGG-3′. The p.Asn770His missense mutation was inserted using the following primers: forward 5′-CCCTAGTCGGGTCGCATGTACCTTGGGCC-3′ and reverse 5′-GGCCCAAGGTACATGCGACCCGACTAGGG-3′. RINGdel is a deletion (amino acids 715-762) of the whole RING finger domain and used as a control (7). Mutations correctly inserted were checked with Sanger sequencing.

### Cell culture and transfection

HEK293T cells (sourced from ATCC) were cultured in Dulbecco’s Modified Eagle Medium/High Glucose (Sigma Aldrich, St Louis, MO, USA) supplemented with 10% fetal bovine serum (Invitrogen, Carlsbad, CA, USA) and 1% penicillin/streptomycin solution (Invitrogen) and incubated at 37°C in a humidified incubator with 5% CO_2_. A total of 300 000 cells/well were seeded in a traditional 6-well plate; after 24 h, cells were transiently transfected using polyethylenimine (PEI 10.5 μl/μg DNA; Sigma Aldrich) with *EAP1* WT and mutated vectors (1 μg/well) generated via site-directed mutagenesis.

### Protein extraction and western blot analysis

Forty-eight hours post-transfection cells were harvested and lysed in radio-immunoprecipitation assay buffer (Sigma Aldrich) supplemented with protease inhibitor (Roche Diagnostics Ltd, West Sussex, UK). The concentration of the cell lysates was measured by the Pierce BCA protein assay kit (Thermo Fischer Scientific) according to the manufacturer’s instructions. Equal amount of proteins was separated by standard deviation score (SDS)-PAGE (4–12% polyacrylamide NuPage Bis–Tris gels; Invitrogen) and transferred on nitrocellulose membranes (Promega, Southampton, UK). Non-specific binding was blocked with 5% non-fat milk in phosphate buffered saline (PBS) containing 0.1% Tween-20 (PBT). Membranes were incubated overnight at 4°C with primary antibodies diluted in 5% non-fat milk in PBT: rabbit polyclonal anti-EAP1 diluted at 1:2000 (Sigma Genosys) and mouse monoclonal anti-GAPDH diluted at 1:5000 (Santa Cruz Biotechnology, Heidelberg, Germany). After washes in PBT, the membranes were probed with secondary antibodies (1:10 000; Licor, Cambridge, UK). After washes in PBT, the membranes were scanned and analyzed using the Odyssey Fc Imaging System (Licor). Experiment was repeated three independent times.

### Promoter assay

HEK293T cells were plated on a 24-well plate (175 000 cells/well) and after 24 h were transiently co-transfected with the same amount of pGL4.10[luc]GnRH vector (7) and SV-40 Renilla (Promega, Southampton, UK) (150 ng/well each) in conjunction with either (i) *EAP1* wild-type or mutant vectors (200 ng/well) or (ii) equal amount of *EAP1* wild-type and p.Ala221del or wild-type and p.Asn770His mutants (100 ng/well each vector). The total amount of DNA transfected was kept constant to 500 ng/well by adding the appropriate amount of pBlueScript vector (i.e. in order to test the basal activation of the signal, co-transfection of pGL4.10[luc]GnRH and SV-40 Renilla only was also performed). Cells were harvested 48 h post-transfection and assayed for luciferase using Dual Luciferase Reporter System (Promega, Southampton, UK) following the manufacturer’s instructions. Each experiment was performed in triplicate and repeated three independent times. Samples were processed using POLARstar Omega microplate reader and data were analyzed using MARS Omega software.

### *In situ* hybridization and immunohistochemistry

Peri-pubertal mouse brains were collected from timed crosses of C57BL/6 mice. Vaginal opening and balanopreputial separation were designated as indicative of the onset of puberty. Brains were fixed in 4% paraformaldehyde (PFA) in PBS, cryoprotected in 30% sucrose in PBS and frozen in OCT compound (VWR); 12 μm thick serial coronal sections were collected on Superfrost Plus slides (VWR). Mouse *Eap1* was PCR-amplified from brain cDNAs using the following primers: *Eap1* FOR: 5′-CAGTCTTGCTACCTGTGCGA-3′ and *Eap1* REV: 5′-AAGCGAGTGGTCCTTCTTGA-3′. Amplified cDNAs were cloned into the dual promoter vector pGEM-T easy (Promega, Southampton, UK) and linearized with the appropriate restriction enzymes. Probe preparation and *in situ* protocol were performed as previously in (40). When co-labeling was desired, after *in situ* hybridization, the sections were incubated with primary antibodies (anti-GnRH; Immunostar, Hudson, Wisconsin, USA) diluted at 1:1000 in PBS–Triton 0.1%, as used in (41). After three washes with PBS–Triton 0.1%, slides were incubated with biotin-conjugated goat secondary antibodies (Vector Laboratories, Peterborough, UK), diluted at 1:300 in PBT and, after further washes, with the avidin–biotin complex (ABC staining kit; Vector Laboratories). The sections were reacted with 3,3′-diaminobenzidine (Vector Laboratories) and mounted in an aqueous compound formed by PBS and glycerol (3:1). Images were acquired using a Leica DM5500B microscope (Leica, Nussloch, Germany), equipped with a DCF295 camera (Leica) and DCViewer software (Leica), and then processed with Abode Photoshop CS6 and Adobe Illustrator CS6.

### Non-human primates

The MBH of female rhesus monkeys (*Macaca mulatta*) was obtained through the Oregon National Primate Research Center Tissue Distribution Program. Animals were classified into different stages of pubertal development following the criteria proposed by Watanabe and Terasawa (42). EJ animals were aged from 9 months to 1.8 years and LJ animals were aged 2–2.9 years. The MBH was collected by making a cut along the posterior border of the optic chiasm, a cut in front of the mammillary bodies, and two lateral cuts half-way between the medial eminence and the hypothalamic sulci, as reported. Tissues were flash frozen in liquid nitrogen and stored at −80°C for later processing.

### ChIP assay

To study the recruitment of EAP1 to the monkey GnRH1 promoter, we performed ChIP assays using chromatin extracted from the MBH of two female rhesus monkeys. One of these animals (1 year and 65 days age) was in the early EJ phase, while the other (2 years and 99 days age) was in the LJ phase of postnatal development. The ChIP procedure was described previously (21,43–45) and was carried out with slight modifications. Tissue cross-linking was performed by incubating the MBH fragments in 1% formaldehyde for 10 min at room temperature. After two additional washing steps in PBS, MBH fragments were lysed with 200 μl SDS buffer (0.5% SDS; 50 mm Tris–HCl; 10 mm EDTA) containing protease, phosphatase and HDAC inhibitors and sonicated for 45 s to yield chromatin fragments of ~500 bp using a Fisher Scientific FB 705 sonicator. Size fragmentation was confirmed by agarose gel electrophoresis. The sonicated chromatin was clarified by centrifugation at 14 000 rpm for 10 min at 4°C, brought up to 1 ml in ChIP dilution buffer (16.7 mm Tris–HCl, pH 8.1, 150 mm NaCl, 1.2 mm EDTA, 1.1% Triton X-100 and 0.01% SDS) containing protease, phosphatase and HDAC inhibitors and stored at −80°C for subsequent immunoprecipitation. For this step, chromatin was pre-cleared with Protein A/G beads (Dynabeads; Invitrogen) for 1 h at 4°C. One hundred microliter aliquots of chromatin were then incubated with 5 μg of the following antibodies: rabbit anti-EAP1 (custom made by Sigma) or anti-beta-galactosidase (Cortex, Biochem, Madison, WI). Antibody–chromatin complexes and 25 μl of protein A beads solution (Dynabeads; ThermoFisher, Waltham, MA) were incubated at 4°C overnight with gentle agitation. Immunocomplexes were washed sequentially with 0.5 ml low-salt wash buffer (20 mm Tris–HCl, pH 8.1, 150 mm NaCl, 2 mm EDTA, 1% Triton X-100 and 0.1% SDS), high-salt wash buffer (20 mm Tris–HCl, pH 8.1, 500 mm NaCl, 2 mm EDTA, 1% Triton X-100 and 0.1% SDS), LiCl buffer (10 mm Tris–HCl, pH 8.1, 250 M LiCl, 1% Nonidet P-40, 1% sodium deoxycholate and 1 mm EDTA) and TE buffer (10 mm Tris–HCl, pH 8.0 and 1 mm EDTA). The immunocomplexes were eluted with 100 μl of 0.1 M NaHCO3 and 1% SDS at 65°C for 45 min. Cross-linking was reversed by adding 4 μl of 5 M NaCl and incubating at 95°C for 30 min. DNA was recovered by using the ChIP DNA clean and concentrator columns (Zymo Research, Irvine, CA) and stored at −80°C until subsequent PCR analysis. All chemicals were purchased from Sigma-Aldrich. Due to the limitation of availability of primate tissue, the experiment was performed only once.

### PCR detection of chromatin-immunoprecipitated DNA

The promoter region of the rhesus monkey GnRH gene (JU472587) was amplified using the forward primer GCCAGAAGCTTCCAGACATCC and the reverse primer AAGTGCAGCCATTAAAACCTCAG. As a negative control for EAP1 binding we used an intergenic region located in intron 2 of the GnRH1 gene (forward primer: ACCACGCCCGGACTGTTTC and reverse primer: TGATCCACTTACCTCGGCTTCC; Eurofins MWG Operon, Huntsville). PCR reactions were performed using 1 μl of each IP and input samples and HotStart Taq Polymerase (Qiagen, Germantown, MD) in a volume of 25 μl. The thermocycling conditions used were 95°C for 5 min, followed by 33 cycles of 15 s at 95°C followed by 30 s at 55°C and 30 s at 72°C. The PCR products were run in a 1.2% agarose gel prepared in Tris/Borate/EDTA buffer.

### Immunofluorescence

Cells were seeded in a 4-well cell culture slide at a density of 1 × 10^5^ cells/well (Millipore, Fisher Scientific, Watford, UK) and transiently transfected with 500 ng/well of WT or mutant Ala221del and Asn770His expression plasmids using PEI. Forty-eight hours after transfection, cells were fixed in 4% PFA in PBS for 15 min and washed with PBS. Samples were permeabilized with 0.1% Triton X-100 in PBS for 30 min and blocked with blocking buffer (10% normal goat serum in PBS) for 30 min. The staining was performed by incubating the samples with rabbit anti-EAP1 diluted at 1:1000 (Sigma Genosys) in blocking buffer for 1 h, followed by a 30 min incubation with goat anti-rabbit Alexa fluor 594 (ThermoFisher Scientific; 1:250) antibody. The cell nuclei were stained with DAPI (Sigma). Images were acquired using a fluorescence microscope (Leica microsystem, Germany) and processed using Adobe Photoshop CS6.

### Statistical analysis

For all experiments, data are expressed as the mean ± SEM. One-way repeated measures analysis of variance was used to determine statistical significance for multiple comparisons; *P*-values (^*^) of <0.05 and (^**^) of <0.01 were considered statistically significant. A *P*-value (^***^) of <0.001 was considered highly significant. The statistical analysis was performed using GraphPad Prism7 (GraphPad software).

## References

[1] Kaprio J., Rimpelä A., Winter T., Viken R.J., Rimpelä M. and Rose R.J. (1995) Common genetic influences on BMI and age at menarche. *Hum. Biol.*, 67, 739–753. 8543288

[2] Juul A., Teilmann G., Scheike T., Hertel N.T., Holm K., Laursen E.M., Main K.M. and Skakkebaek N.E. (2006) Pubertal development in Danish children: comparison of recent European and US data. *Int. J. Androl.*, 29, 247, 286–255; discussion, 290. 16466546 10.1111/j.1365-2605.2005.00556.x

[3] Teilmann G., Pedersen C.B., Skakkebaek N.E. and Jensen T.K. (2006) Increased risk of precocious puberty in internationally adopted children in Denmark. *Pediatrics*, 118, e391–e399. 16882780 10.1542/peds.2005-2939

[4] Parent A.S., Teilmann G., Juul A., Skakkebaek N.E., Toppari J. and Bourguignon J.P. (2003) The timing of normal puberty and the age limits of sexual precocity: variations around the world, secular trends, and changes after migration. *Endocr. Rev.*, 24, 668–693. 14570750 10.1210/er.2002-0019

[5] Morris D.H., Jones M.E., Schoemaker M.J., Ashworth A. and Swerdlow A.J. (2011) Familial concordance for age at menarche: analyses from the Breakthrough Generations Study. *Paediatr. Perinat. Epidemiol.*, 25, 306–311. 21470270 10.1111/j.1365-3016.2010.01183.x

[6] Rampazzo A., Pivotto F., Occhi G., Tiso N., Bortoluzzi S., Rowen L., Hood L., Nava A. and Danieli G.A. (2000) Characterization of C14orf4, a novel intronless human gene containing a polyglutamine repeat, mapped to the ARVD1 critical region. *Biochem. Biophys. Res. Commun.*, 278, 766–774. 11095982 10.1006/bbrc.2000.3883

[7] Heger S., Mastronardi C., Dissen G.A., Lomniczi A., Cabrera R., Roth C.L., Jung H., Galimi F., Sippell W. and Ojeda S.R. (2007) Enhanced at puberty 1 (EAP1) is a new transcriptional regulator of the female neuroendocrine reproductive axis. *J. Clin. Invest.*, 117, 2145–2154. 17627301 10.1172/JCI31752 PMC1906733

[8] Dissen G.A., Lomniczi A., Heger S., Neff T.L. and Ojeda S.R. (2012) Hypothalamic EAP1 (enhanced at puberty 1) is required for menstrual cyclicity in nonhuman primates. *Endocrinology*, 153, 350–361. 22128022 10.1210/en.2011-1541 PMC3249687

[9] Li C. and Li P. (2017) Enhanced at puberty-1 (Eap1) expression critically regulates the onset of puberty independent of hypothalamic Kiss1 expression. *Cell. Physiol. Biochem.*, 43, 1402–1412. 29017168 10.1159/000481872

[10] Matagne V., Mastronardi C., Shapiro R.A., Dorsa D.M. and Ojeda S.R. (2009) Hypothalamic expression of Eap1 is not directly controlled by ovarian steroids. *Endocrinology*, 150, 1870–1878. 19022886 10.1210/en.2008-0779 PMC2659281

[11] Palmert M.R. and Dunkel L. (2012) Clinical practice. Delayed puberty. *N. Engl. J. Med.*, 366, 443–453. 22296078 10.1056/NEJMcp1109290

[12] Zhu J. and Chan Y.M. (2017) Adult consequences of self-limited delayed puberty. *Pediatrics*, 139. 10.1542/peds.2016-3177 PMC8579478 28562264

[13] Howard S.R. and Dunkel L. (2018) The genetic basis of delayed puberty. *Neuroendocrinology*, 106, 283–291. 28926843 10.1159/000481569

[14] Wehkalampi K., Widén E., Laine T., Palotie A. and Dunkel L. (2008) Patterns of inheritance of constitutional delay of growth and puberty in families of adolescent girls and boys referred to specialist pediatric care. *J. Clin. Endocrinol. Metab.*, 93, 723–728. 18160460 10.1210/jc.2007-1786

[15] Howard S.R., Guasti L., Ruiz-Babot G., Mancini A., David A., Storr H.L., Metherell L.A., Sternberg M.J., Cabrera C.P., Warren H.R. et al. (2016) IGSF10 mutations dysregulate gonadotropin-releasing hormone neuronal migration resulting in delayed puberty. *EMBO Mol. Med.*, 8, 626–642. 27137492 10.15252/emmm.201606250 PMC4888853

[16] Howard S.R., Oleari R., Poliandri A., Chantzara V., Fantin A., Ruiz-Babot G., Metherell L.A., Cabrera C.P., Barnes M.R., Wehkalampi K. et al. (2018) HS6ST1 insufficiency causes self-limited delayed puberty in contrast with other GnRH deficiency genes. *J. Clin. Endocrinol. Metab.*, 103, 3420–3429. 29931354 10.1210/jc.2018-00646 PMC6126894

[17] Benjamini Y., Drai D., Elmer G., Kafkafi N. and Golani I. (2001) Controlling the false discovery rate in behavior genetics research. *Behav. Brain Res.*, 125, 279–284. 11682119 10.1016/s0166-4328(01)00297-2

[18] Ojeda S.R., Lomniczi A., Mastronardi C., Heger S., Roth C., Parent A.S., Matagne V. and Mungenast A.E. (2006) Minireview: the neuroendocrine regulation of puberty: is the time ripe for a systems biology approach? *Endocrinology*, 147, 1166–1174. 16373420 10.1210/en.2005-1136

[19] Plant T.M. (2015) Neuroendocrine control of the onset of puberty. *Front. Neuroendocrinol.*, 38, 73–88. 25913220 10.1016/j.yfrne.2015.04.002 PMC4457677

[20] Ojeda S.R., Lomniczi A., Loche A., Matagne V., Kaidar G., Sandau U.S. and Dissen G.A. (2010) The transcriptional control of female puberty. *Brain Res.*, 1364, 164–174. 20851111 10.1016/j.brainres.2010.09.039 PMC2992593

[21] Lomniczi A., Wright H., Castellano J.M., Matagne V., Toro C.A., Ramaswamy S., Plant T.M. and Ojeda S.R. (2015) Epigenetic regulation of puberty via zinc finger protein-mediated transcriptional repression. *Nat. Commun.*, 6, 10195. 26671628 10.1038/ncomms10195 PMC4703871

[22] Sedlmeyer I.L., Hirschhorn J.N., Palmert M.R. (2002) Pedigree analysis of constitutional delay of growth and maturation: determination of familial aggregation and inheritance patterns. *J. Clin. Endocrinol. Metab.*, 87, 5581–5586. 12466356 10.1210/jc.2002-020862

[23] Marcogliese P.C., Shashi V., Spillmann R.C., Stong N., Rosenfeld J.A., Koenig M.K., Martinez-Agosto J.A., Herzog M., Chen A.H., Dickson P.I. et al. (2018) IRF2BPL is associated with neurological phenotypes. *Am. J. Hum. Genet.*, 103, 456. 10.1016/j.ajhg.2018.08.010 PMC6128320 30193138

[24] Lomniczi A., Garcia-Rudaz C., Ramakrishnan R., Wilmot B., Khouangsathiene S., Ferguson B., Dissen G.A. and Ojeda S.R. (2012) A single-nucleotide polymorphism in the EAP1 gene is associated with amenorrhea/oligomenorrhea in nonhuman primates. *Endocrinology*, 153, 339–349. 22128021 10.1210/en.2011-1540 PMC3249686

[25] Mueller J.K., Dietzel A., Lomniczi A., Loche A., Tefs K., Kiess W., Danne T., Ojeda S.R. and Heger S. (2011) Transcriptional regulation of the human KiSS1 gene. *Mol. Cell. Endocrinol.*, 342, 8–19. 21672609 10.1016/j.mce.2011.04.025 PMC3148268

[26] Moreira-Andrés M.N., Cañizo F.J., Cruz F.J. de la, Gómez-de la Cámara A. and Hawkins F.G. (1998) Bone mineral status in prepubertal children with constitutional delay of growth and puberty. *Eur. J. Endocrinol.*, 139, 271–275. 9758435 10.1530/eje.0.1390271

[27] Finkelstein J.S., Neer R.M., Biller B.M., Crawford J.D. and Klibanski A. (1992) Osteopenia in men with a history of delayed puberty. *N. Engl. J. Med.*, 326, 600–604. 1734250 10.1056/NEJM199202273260904

[28] Crowne E.C. and Shalet S.M. (1990) Management of constitutional delay in growth and puberty. *Trends Endocrinol. Metab.*, 1, 239–242. 18411125 10.1016/1043-2760(90)90003-l

[29] Kaltiala-Heino R., Kosunen E. and Rimpelä M. (2003) Pubertal timing, sexual behaviour and self-reported depression in middle adolescence. *J. Adolesc.*, 26, 531–545. 12972267 10.1016/s0140-1971(03)00053-8

[30] Day F.R., Elks C.E., Murray A., Ong K.K. and Perry J.R. (2015) Puberty timing associated with diabetes, cardiovascular disease and also diverse health outcomes in men and women: the UK Biobank study. *Sci. Rep.*, 5, 11208. 26084728 10.1038/srep11208 PMC4471670

[31] Topaloglu A.K., Tello J.A., Kotan L.D., Ozbek M.N., Yilmaz M.B., Erdogan S., Gurbuz F., Temiz F., Millar R.P. and Yuksel B. (2012) Inactivating KISS1 mutation and hypogonadotropic hypogonadism. *N. Engl. J. Med.*, 366, 629–635. 22335740 10.1056/NEJMoa1111184

[32] Tusset C., Noel S.D., Trarbach E.B., Silveira L.F., Jorge A.A., Brito V.N., Cukier P., Seminara S.B., Mendonça B.B., Kaiser U.B. and Latronico A.C. (2012) Mutational analysis of TAC3 and TACR3 genes in patients with idiopathic central pubertal disorders. *Arq. Bras. Endocrinol. Metabol.*, 56, 646–652. 23329188 10.1590/s0004-27302012000900008 PMC3712828

[33] Zhu J., Choa R.E., Guo M.H., Plummer L., Buck C., Palmert M.R., Hirschhorn J.N., Seminara S.B. and Chan Y.M. (2015) A shared genetic basis for self-limited delayed puberty and idiopathic hypogonadotropic hypogonadism. *J. Clin. Endocrinol. Metab.*, 100, 646–654. 10.1210/jc.2015-1080 PMC4399304 25636053

[34] Wehkalampi K., Widén E., Laine T., Palotie A. and Dunkel L. (2008) Association of the timing of puberty with a chromosome 2 locus. *J. Clin. Endocrinol. Metab.*, 93, 4833–4839. 18812480 10.1210/jc.2008-0882 PMC2685475

[35] Wang K., Li M. and Hakonarson H. (2010) ANNOVAR: functional annotation of genetic variants from high-throughput sequencing data. *Nucleic Acids Res.*, 38, e164. 20601685 10.1093/nar/gkq603 PMC2938201

[36] Adzhubei I.A., Schmidt S., Peshkin L., Ramensky V.E., Gerasimova A., Bork P., Kondrashov A.S. and Sunyaev S.R. (2010) A method and server for predicting damaging missense mutations. *Nat. Methods*, 7, 248–249. 20354512 10.1038/nmeth0410-248 PMC2855889

[37] Kumar P., Henikoff S. and Ng P.C. (2009) Predicting the effects of coding non-synonymous variants on protein function using the SIFT algorithm. *Nat. Protoc.*, 4, 1073–1081. 19561590 10.1038/nprot.2009.86

[38] Kelley L.A., Mezulis S., Yates C.M., Wass M.N. and Sternberg M.J. (2015) The Phyre2 web portal for protein modeling, prediction and analysis. *Nat. Protoc.*, 10, 845–858. 25950237 10.1038/nprot.2015.053 PMC5298202

[39] Jones D.T. and Cozzetto D. (2015) DISOPRED3: precise disordered region predictions with annotated protein-binding activity. *Bioinformatics*, 31, 857–863. 25391399 10.1093/bioinformatics/btu744 PMC4380029

[40] Guasti L., Paul A., Laufer E. and King P. (2011) Localization of Sonic hedgehog secreting and receiving cells in the developing and adult rat adrenal cortex. *Mol. Cell. Endocrinol.*, 336, 117–122. 21094676 10.1016/j.mce.2010.11.010 PMC3063526

[41] Cariboni A., Davidson K., Rakic S., Maggi R., Parnavelas J.G. and Ruhrberg C. (2011) Defective gonadotropin-releasing hormone neuron migration in mice lacking SEMA3A signalling through NRP1 and NRP2: implications for the aetiology of hypogonadotropic hypogonadism. *Hum. Mol. Genet.*, 20, 336–344. 21059704 10.1093/hmg/ddq468

[42] Watanabe G. and Terasawa E. (1989) *In vivo*release of luteinizing hormone releasing hormone increases with puberty in the female rhesus monkey. *Endocrinology*, 125, 92–99. 2661213 10.1210/endo-125-1-92

[43] Lomniczi A., Loche A., Castellano J.M., Ronnekleiv O.K., Bosch M., Kaidar G., Knoll J.G., Wright H., Pfeifer G.P. and Ojeda S.R. (2013) Epigenetic control of female puberty. *Nat. Neurosci.*, 16, 281–289. 23354331 10.1038/nn.3319 PMC3581714

[44] Toro C.A., Wright H., Aylwin C.F., Ojeda S.R. and Lomniczi A. (2018) Trithorax dependent changes in chromatin landscape at enhancer and promoter regions drive female puberty. *Nat. Commun.*, 9, 57. 29302059 10.1038/s41467-017-02512-1 PMC5754362

[45] Vazquez M.J., Toro C.A., Castellano J.M., Ruiz-Pino F., Roa J., Beiroa D., Heras V., Velasco I., Dieguez C., Pinilla L. et al. (2018) SIRT1 mediates obesity- and nutrient-dependent perturbation of pubertal timing by epigenetically controlling Kiss1 expression. *Nat. Commun.*, 9, 4194. 30305620 10.1038/s41467-018-06459-9 PMC6179991

[46] Schwarz J.M., Cooper D.N., Schuelke M. and Seelow D. (2014) MutationTaster2: mutation prediction for the deep-sequencing age. *Nat. Methods*, 11, 361–362. 24681721 10.1038/nmeth.2890

